# Efficacy of Protein and Essential Amino Acid Supplementation in Lower Limb Surgeries: A Systematic Review and Meta-Analysis

**DOI:** 10.7759/cureus.69212

**Published:** 2024-09-11

**Authors:** Akikazu Hagiyama, Norio Yamamoto, Jun Watanabe, Takahiro Tsuge, Yuki Nakashima, Shuri Nakao, Hiroki Sato, Takashi Yorifuji

**Affiliations:** 1 Division of Physical Medicine and Rehabilitation, Okayama University Hospital, Okayama, JPN; 2 Department of Epidemiology, Graduate School of Medicine, Dentistry and Pharmaceutical Sciences, Okayama University, Okayama, JPN; 3 Department of Systematic Reviewers, Scientific Research WorkS Peer Support Group, Osaka, JPN; 4 Department of Surgery, Division of Gastroenterological, General and Transplant Surgery, Jichi Medical University, Shimotsuke, JPN; 5 Center for Community Medicine, Jichi Medical University, Shimotsuke, JPN; 6 Department of Rehabilitation, Kurashiki Medical Center, Kurashiki, JPN; 7 Department of Clinical Practice and Support, Division of Rehabilitation, Hiroshima University Hospital, Hiroshima, JPN; 8 Division of Rehabilitation Medicine, Shimane University Hospital, Izumo, JPN; 9 Department of Physical Therapy, Faculty of Rehabilitation, Kawasaki University of Medical Welfare, Kurashiki, JPN; 10 Department of Radiological Technology, Graduate School of Health Sciences, Okayama University, Okayama, JPN

**Keywords:** essential amino acid, lower limb surgery, meta-analysis, protein, systematic review

## Abstract

This study aimed to examine the efficacy and safety of protein and/or essential amino acid (EAA) supplementation in all lower limb surgeries using systematic reviews and meta-analysis of randomized controlled trials (RCTs). We included RCTs that assessed the efficacy of protein and/or EAA supplementation in lower limb surgeries. On June 2, 2023, we searched EMBASE, MEDLINE, the Cochrane Central Register of Controlled Trials, the World Health Organization International Clinical Trials Registry Platform, and ClinicalTrials.gov. The primary outcomes were mobility, patient-reported outcomes (PRO), and acute kidney injury (AKI). The secondary outcomes were exercise capacity, muscle strength, muscle mass, and all adverse events. We performed meta-analyses using the random-effects model. We assessed the risk of bias using the Cochrane risk-of-bias tool and the certainty of evidence using the Grading of Recommendations, Assessment, Development, and Evaluation approach. We included 12 RCTs (622 patients). These studies included four on hip fracture surgery, three on total hip arthroplasty, and five on total knee arthroplasty. Protein and/or EAA supplementation may slightly improve PRO (standard mean difference 0.51, 95% confidence interval (CI): 0.22 to 0.80, low certainty of evidence). Nevertheless, it may not improve mobility (mean difference 0.07 m/s, 95% CI: -0.01 to 0.16, low certainty of evidence). No adverse events including AKI were reported. Muscle strength may have increased (standard mean difference 0.31, 95% CI: 0.02 to 0.61, very low certainty of evidence). However, exercise capacity (mean difference 5.43 m, 95% CI: -35.59 to 46.45, very low certainty of evidence) and muscle mass (standard mean difference -0.08, 95% CI: -0.49 to 0.33, very low certainty of evidence) were not improved. While protein and/or EAA supplementation in lower limb surgeries may improve PRO, it is unlikely to affect mobility. Despite this, the medical team and patients might still consider protein and/or EAA supplementation a useful option.

## Introduction and background

Perioperative immobilization and surgical stress related to lower limb surgeries, such as fracture fixation, arthroplasty, and ligament reconstruction, can lead to atrophy and weakness of the lower limb muscles, resulting in decreased mobility [1-3]. Despite improvements in pain and gait ability, patients after total knee arthroplasty (TKA) remain about 39% weaker in quadriceps muscle strength and 17% slower in gait speed than healthy people even one year after surgery [4]. This decreased mobility can negatively impact the quality of life (QOL) and patient-reported outcomes (PRO) [5,6]. Despite advances in surgical techniques and postoperative care, muscle atrophy and weakness after lower limb surgeries remain common. 

There is an increasing interest in nutritional interventions to reduce postoperative muscle atrophy and weakness in surgery, including lower limb surgeries [7]. Surgical stress and perioperative immobilization can accelerate catabolism and proteolysis in skeletal muscles [7]. When protein intake is insufficient, skeletal muscle becomes the primary source of essential amino acid (EAA) to maintain protein synthesis, which can lead to further muscle atrophy [7]. Therefore, there is an increased need for postoperative protein intake to satisfy the body’s elevated EAA demands and mitigate muscle catabolism [7]. During postoperative rehabilitation, it is recommended that protein intake be at least 1.6 g/kg/day and up to 2.0-3.0 g/kg/day [8,9]. The effectiveness of protein intake in stimulating muscle protein synthesis primarily depends on EAA, which is essential for muscle repair and growth [9]. Furthermore, sufficient protein intake before surgery is also crucial in addition to postoperative care [7]. Providing sufficient protein intake before and after surgery can better prepare the patient for the surgical procedure and enhance the effectiveness of postoperative rehabilitation. Previous randomized controlled trials (RCTs) have reported that protein and/or EAA supplementation prevents muscle weakness and decreased mobility after various types of lower limb surgeries, including hip fracture, total hip arthroplasty (THA), and TKA [10-12].

Previous systematic reviews (SRs) focused on specific lower limb surgeries, making unclear the overall effects of protein and/or EAA supplementation in all lower limb surgeries and these variations across different surgeries [13-16]. Furthermore, earlier SRs suggested potential advantages of protein and/or EAA supplementation after various lower limb surgeries, but their quantitative impact remains unclear due to the absence of meta-analysis [15-17]. Moreover, in the absence of SRs investigating adverse events in all lower limb surgeries, examining the safety of protein and/or EAA supplementation is essential. 

Therefore, our study aimed to examine the efficacy and safety of protein and/or EAA supplementation in all lower limb surgeries using SRs and meta-analysis.

## Review

Methods

This SR and meta-analysis followed the Cochrane Handbook and the Preferred Reporting Items for Systematic Reviews and Meta-analyses 2020 (PRISMA 2020) guideline (Appendix Table 3) [18,19]. This protocol was registered with the Open Science Forum (https://osf.io/u23w8/).

The inclusion criteria for the reviewed articles were RCTs that assessed the efficacy of protein and/or EAA supplementation in lower limb surgeries. We included all papers, including published and unpublished articles, abstracts of conferences, and letters. We did not apply restrictions on language, country, observation period, and publication year.

We included patients who underwent all lower limb surgeries without specifying age and sex. We included both inpatients and outpatients. The exclusion criteria were studies conducted on animal models.

The intervention was additional dietary protein and/or EAA supplementation to augment the usual dietary protein intake. Based on previous studies, we defined the intervention for nutritional therapies not explicitly labeled as protein or EAA if more than 25% of the total energy intake was derived from protein and/or EAA [20,21]. The intervention was provided alone or in combination with usual exercise training. The control was placebo or usual care.

We selected the primary and secondary outcomes based on core outcomes reported in studies of major lower limb surgeries [22-24]. The outcomes were prioritized according to their clinical importance. The primary outcomes were as follows: 

Mobility

Mobility was defined as gait speed measured by short-distance gaiting tests or Timed Up & Go test (TUG). Gait speed was recorded in meters per second (m/s). When TUG was reported, we converted the results to gait speed based on the previous study [25]. We prioritized gait speed measured by short-distance gaiting tests if several mobility outcomes were measured in the same study. 

PRO

PRO refers to the self-reported health status directly associated with a particular lower limb disease or condition. These outcomes were measured using scales such as the Harris Hip Score (HHS), Knee injury and Osteoarthritis Outcome Score (KOOS), Western Ontario and McMaster Universities Osteoarthritis Index (WOMAC), and Oxford Knee Score (OKS) [26,27]. We also included other scales if they were used in the original studies.

Acute Kidney Injury (AKI)

We defined AKI as a major adverse event because previous studies have raised concerns that high protein and/or EAA intake might lead to kidney dysfunction [20,28]. AKI was defined as a sudden loss of excretory kidney function, identified by a rapid increase in serum creatinine and a decrease in urine output [29]. We also included other definitions of AKI if they were used in the original studies. The secondary outcomes were as follows:

Exercise Capacity

We defined exercise capacity as the distance measured by a 6-minute walk test (6MWT) and was recorded in meters (m). 

Muscle Strength

We defined muscle strength as the quadriceps strength on the operative side. We included all measurement methods and units in our analysis.

Muscle Mass

We defined muscle mass as the cross-sectional area and volume of the quadriceps on the operative side measured using computed tomography (CT), magnetic resonance imaging (MRI), and ultrasound (US). 

All Adverse Events

We defined all adverse events such as death, cardiovascular events, allergic reactions, gastrointestinal dysfunction, kidney dysfunction including AKI, and liver dysfunction. We also included other definitions of adverse events if they were used in the original studies. We counted the incidence of these events. 

Regarding mobility, PRO, exercise capacity, muscle strength, and muscle mass, we used data between two weeks and six months post-surgery. We selected the data point closest to six months for analysis when multiple measurements were reported. Regarding AKI and all adverse events, we recorded them during the entire follow-up period in each study.

Search Strategy and Selection of Studies

On June 2, 2023, we searched EMBASE via Dialog, MEDLINE via PubMed, the Cochrane Central Register of Controlled Trials (CENTRAL), the World Health Organization International Clinical Trials Registry Platform (ICTRP), and ClinicalTrials.gov. to identify all relevant RCTs. Details of search strategies are shown in Appendix Table 4. Pairs from five reviewers (AH, TT, YN, SN, and HS) independently screened the titles and abstracts of the search results to determine whether each study fulfilled the inclusion criteria. Pairs from five reviewers (AH, TT, YN, SN, and HS) independently performed full-text reviews to assess the eligibility of each study. Disagreements were resolved by consultation with a third reviewer (NY). We checked the reference lists of studies, including international guidelines, and the reference lists of eligible studies and articles citing eligible studies [30]. We asked the authors of original studies for unpublished or additional data if these studies lacked the required information. 

Data Extraction and Quality Assessment

Pairs from five reviewers (AH, TT, YN, SN, and HS) independently performed data extraction of the included studies using prespecified forms. Extracted data included participant characteristics (e.g., age, sex, and type of surgery), intervention details (e.g., type, timing, and duration), outcomes (e.g., mobility and PRO), comparator (placebo and standard care), and funding presence. We contacted the authors of these studies if needed more information. Pairs from five reviewers (AH, TT, YN, SN, and HS) independently assessed the risk of bias using version 2 of the Cochrane risk-of-bias tool for randomized trials (RoB 2) [31]. Any disagreements in data extraction or risk of bias assessment were resolved by consultation with a third reviewer (NY).

Data Analyses

All analyses were performed using RevMan software version 5.4.1 (The Nordic Cochrane Center, The Cochrane Collaboration, Copenhagen, Denmark). We used a random-effects model for all the meta-analyses. We pooled the mean differences (MDs) and the 95% confidence intervals (CIs) for mobility and exercise capacity. We pooled the standard mean differences (SMDs) and 95% CIs for PRO, muscle strength, and muscle mass. We pooled the relative risk ratios and the 95% CIs for AKI and all adverse events.

We performed an intention-to-treat analysis for all dichotomous variables to handle missing data. For continuous variables, we did not impute missing data, as recommended by the Cochrane Handbook [18].

To assess heterogeneity, we inspected forest plots visually and calculated the I^2^ statistic to evaluate statistical heterogeneity. The interpretation of I^2^ values was as follows: 0-40% might not be important; 30-60% may represent moderate heterogeneity; 50-90% may represent substantial heterogeneity; and 75-100% considerable heterogeneity. We investigated the underlying reasons in cases with substantial heterogeneity (I^2^ > 50%). The statistical significance of heterogeneity was determined using the Cochrane Chi^2^ test (Q-test) for the I^2^ statistic, with a p-value of <0.10 [18].

To assess reporting bias, we searched the clinical trials registration system (ClinicalTrials.gov and ICTRP) and performed an extensive literature search for unpublished trials. To assess outcome reporting bias, we compared the outcomes defined in trial protocols with those reported in the publications. 

The following prespecified subgroup analyses of the primary and secondary outcomes were planned to determine the potential causes of heterogeneity: (i) Age group: Under 18 years versus 18 to 64 years versus 65 years and over; (ii) Surgery type: Elective surgery versus non-elective surgery; (iii) Surgical area: Hip joint and surrounding areas versus knee joint and surrounding areas versus ankle joint and surrounding areas; (iv) Surgical technique: Hip fracture surgery versus total hip arthroplasty versus total knee arthroplasty versus anterior cruciate ligament reconstruction versus Achilles tendon repair; (v) Supplementation type: Protein versus EAA versus the combination of protein and EAA; (vi) Intervention timing: Initiated within six weeks after surgery versus after six weeks [32]; (vii) Intervention duration: Less than four weeks versus more than four weeks; (viii) Exercise intensity: Protein and/or EAA supplementation with standard exercise therapy versus protein and/or EAA supplementation with intensive exercise therapy

The following prespecified sensitivity analyses of the primary and secondary outcomes were planned: (i) Exclusion of studies using imputed statistics; (ii) Only the participants who complete the study with complete data; (iii) AKI as defined by clinical practice guidelines only [29].

We presented the summary of findings for the primary and secondary outcomes following the Cochrane Handbook, including grading the evidence for each outcome using the Grading of Recommendations, Assessment, Development, and Evaluation (GRADE) approach [18]. The certainty of evidence using the GRADE approach was evaluated by two reviewers (AH and NY). Any disagreements between the two reviewers were resolved by consultation with a third reviewer (JW).

Difference Between the Protocol and Review

We included an additional criterion for defining interventions in nutritional therapies not explicitly labeled as protein or EAA. We defined interventions in our study as those involving more than 25% of total energy intake from protein and/or EAA [20,21].

Results

Search Results

**Figure 1:**
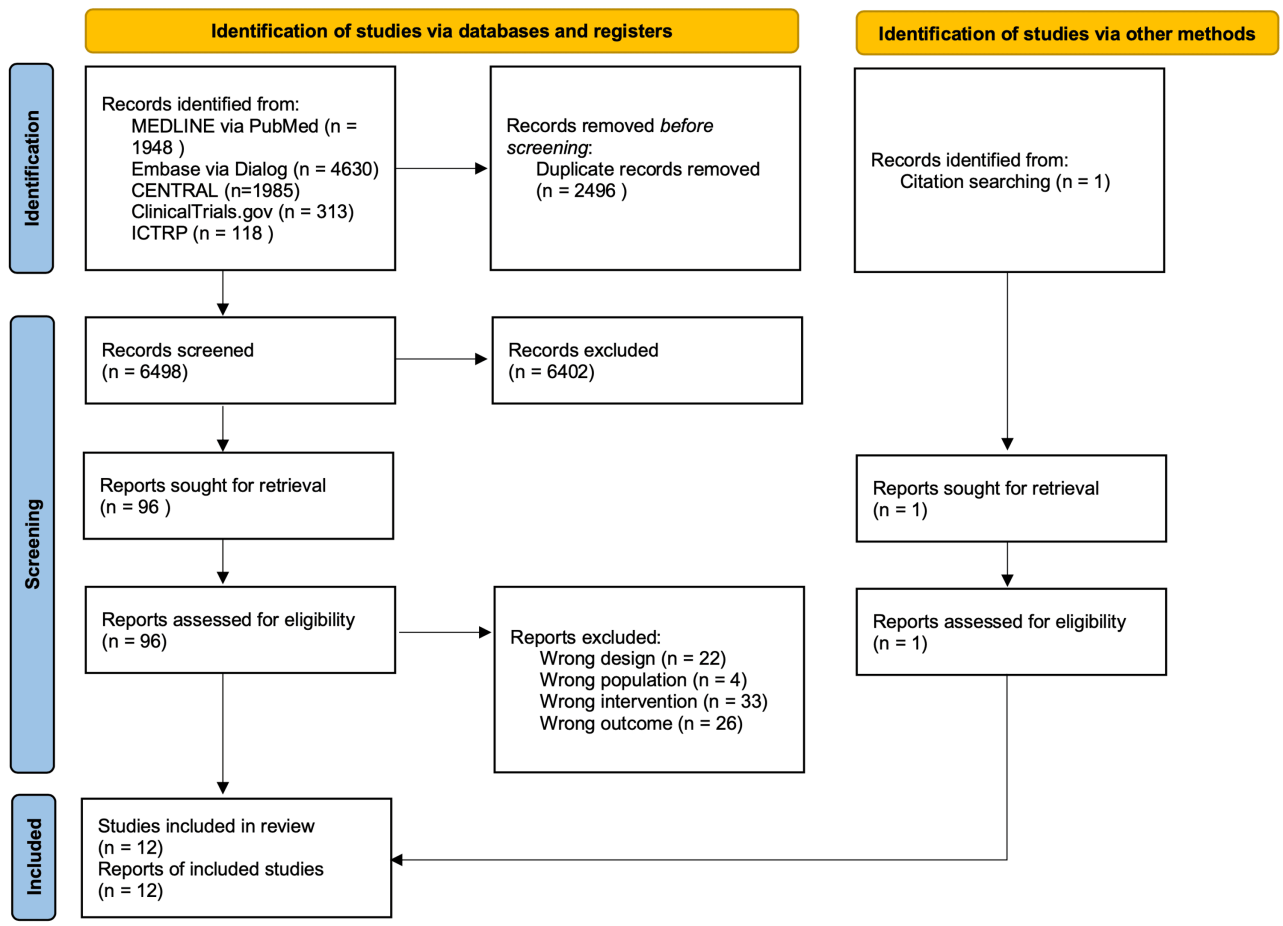
PRISMA study flow diagram PRISMA, Preferred Reporting Items for Systematic Reviews and Meta-Analyses

After excluding duplicates, 6498 studies were identified. We excluded 85 studies after reviewing the full texts for eligibility (Appendix Table 5). Finally, we included 12 studies in the synthesis (Figure 1) [10-12,33-41]. Table 1 shows the characteristics of the included studies. A total of 622 patients were included. The studies included four on hip fracture surgery, three on THA, and five on TKA. The supplementations were protein in two studies, EAA in nine studies, and the combination of protein and EAA in one study. The follow-up period was from two weeks to two years.

**Table 1:** Characteristics of included studies TKA, total knee arthroplasty; THA, total hip arthroplasty; EAA, essential amino acid; SD, standard deviation

Author	Year	Country	Type of surgery	Number of patients (n) (Intervention/Control)	Age (Mean [SD])	Sex (Male/Female, %)	Type of supplementation	Control	Intervention timing	Intervention duration	Follow-up period after surgery	Funding
													Dreyer et al. [33]	2013	USA	TKA	40 (21/19)	69.1 (5.1)	32.1/67.9	EAA	Placebo	1 week pre to 2 weeks post surgery	3 weeks	6 weeks	Yes
Niitsu et al. [34]	2016	Japan	Hip fracture surgery	38 (20/18)	79.7 (8.1)	0.0/100.0	Protein	Standard care	1 day to 2 weeks after surgery	2 weeks	2 weeks	Yes
Baldissarro et al. [35]	2016	Italy	THA	60 (30/30)	66.6 (8.4)	40.0/60.0	EAA	Placebo	3-5 weeks after surgery	2 weeks	2 weeks	Not stated
Dreyer et al. [36]	2018	USA	TKA	67 (36/31)	64.4 (5.9)	35.9/64.1	EAA	Placebo	1 week pre to 6 weeks post surgery	7 weeks	6 weeks	Yes
Ikeda et al. [37]	2019	Japan	THA	31 (18/13)	75.4 (5.7)	0.0/100.0	EAA	Placebo	Post surgery (details not shown)	3.4 weeks	3.1 weeks	Not stated
Invernizzi et al. [38]	2019	Italy	Hip fracture surgery	32 (16/16)	79.0 (7.8)	15.6/84.4	EAA	Standard care	3 months after fracture	8.7 weeks	8 weeks	Not stated
Aquilani et al. [11]	2019	Italy	Hip fracture surgery	56 (28/28)	80.8 (7.3)	39.3/60.7	EAA	Placebo	20±11 days after fracture	5.7 weeks	8.6 weeks	Not stated
Ueyama et al. [39]	2020	Japan	TKA	60 (30/30)	75.9 (7.5)	16.7/83.3	EAA	Placebo	1 week pre to 2 weeks post surgery	3 weeks	4 weeks	No
Ninomiya et al. [12]	2022	Japan	THA	58 (29/29)	70.6 (3.5)	0.0/100.0	Protein&EAA	Standard care	4 weeks pre to 8 weeks post surgery	12 weeks	12 weeks	Yes
Nie et al. [40]	2023	China	Hip fracture surgery	100 (50/50)	67.0 (5.1)	53.0/47.0	Protein	Standard care	10 days to 3 months and 10 days after surgery	12.9 weeks	16 weeks	Yes
Ueyama et al. [10]	2023	Japan	TKA	60 (30/30)	75.8 (7.1)	19.2/80.8	EAA	Placebo	1 week pre to 2 weeks post surgery	3 weeks	104.3 weeks	No
Pandor et al. [41]	2023	India	TKA	20 (10/10)	64.4 (4.2)	55.0/45.0	EAA	Placebo	1 week pre to 6 weeks post surgery	7 weeks	6 weeks	No

Risk of Bias Assessments

**Figure 2:**
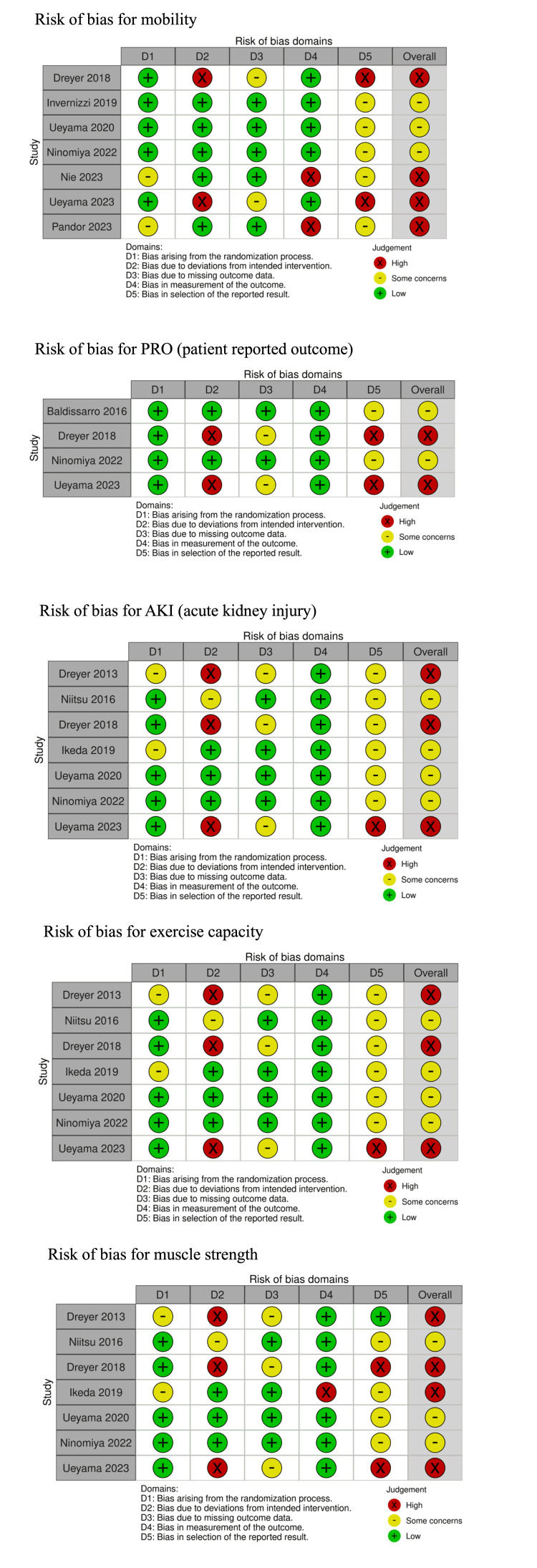
Risk of bias PRO, patient-reported outcomes; AKI, acute kidney injury

The risk of bias assessments for each study is shown in Figure 2. The overall risk of bias in all outcomes was of some concern or high, mainly due to most studies lacking preregistered protocols or predefined analysis plans.

Primary Outcomes

Table 2 shows the summary of findings of this review.

**Table 2:** Summary of findings Patient: Patients who underwent lower limb surgery Setting: Patients who underwent all lower limb surgery without specifying age and sex Intervention: Protein and EAA supplementation Comparison: Control †The risk in the intervention group (and its 95% confidence interval) is based on the assumed risk in the comparison group and the relative effect of the intervention (and its 95% CI). AKI: acute kidney injury; CI: confidence interval; EAA: essential amino acid; MD: mean difference; PRO: patient-reported outcomes; SMD: standardized mean difference GRADE Working Group grades of evidence High certainty: we are very confident that the true effect lies close to that of the estimate of the effect. Moderate certainty: we are moderately confident in the effect estimate: the true effect is likely to be close to the estimate of the effect, but there is a possibility that it is substantially different. Low certainty: our confidence in the effect estimate is limited: the true effect may be substantially different from the estimate of the effect. Very low certainty: we have very little confidence in the effect estimate: the true effect is likely to be substantially different from the estimate of effect. a Downgraded one level because of risk of bias b Downgraded one level because of imprecision c Downgraded two levels because of imprecision

Outcomes	Anticipated absolute effects† (95% CI)		Relative effect (95% CI)	№ of participants (studies)	Certainty of the evidence (GRADE)	Comments
	Risk with control	Risk with Protein and EAA supplementation				
Mobility	The mean mobility ranged from 0.63 to 1.08 m/s	MD 0.07 m/s higher (0.01 lower to 0.16 higher)	-	361 (7 RCTs)	Low a, b	Protein and/or EAA may result in little to no difference in mobility
PRO	-	SMD 0.51 higher (0.22 higher to 0.8 higher)	-	209 (4 RCTs)	Low a, b	Protein and/or EAA may slightly improve PRO
AKI	The incidence of AKI was 0.0% in both the control group and the protein and EAA supplementation group		not estimable	354 (7 RCTs)	Low a, b	Protein and/or EAA may not cause AKI
Exercise capacity	The mean exercise capacity ranged from 145.8 to 477.02 m	MD 5.43 m higher (35.59 lower to 46.45 higher)	-	95 (2 RCTs)	Very low a, c	The evidence is very uncertain about the effect of protein and/or EAA on exercise capacity
Muscle strength	-	SMD 0.31 higher (0.02 higher to 0.61 higher)	-	306 (7 RCTs)	Very low a, c	Protein and/or EAA may increase muscle strength but the evidence is very uncertain
Muscle mass	-	SMD 0.08 lower (0.49 lower to 0.33 higher)	-	179 (4 RCTs)	Very low a, c	The evidence is very uncertain about the effect of protein and/or EAA on muscle mass
All adverse events	The incidence of all adverse events was 0.0% in both the control group and the protein and EAA supplementation group		not estimable	354 (7 RCTs)	Low a, b	Protein and/or EAA may not cause all adverse events

Mobility

**Figure 3:**
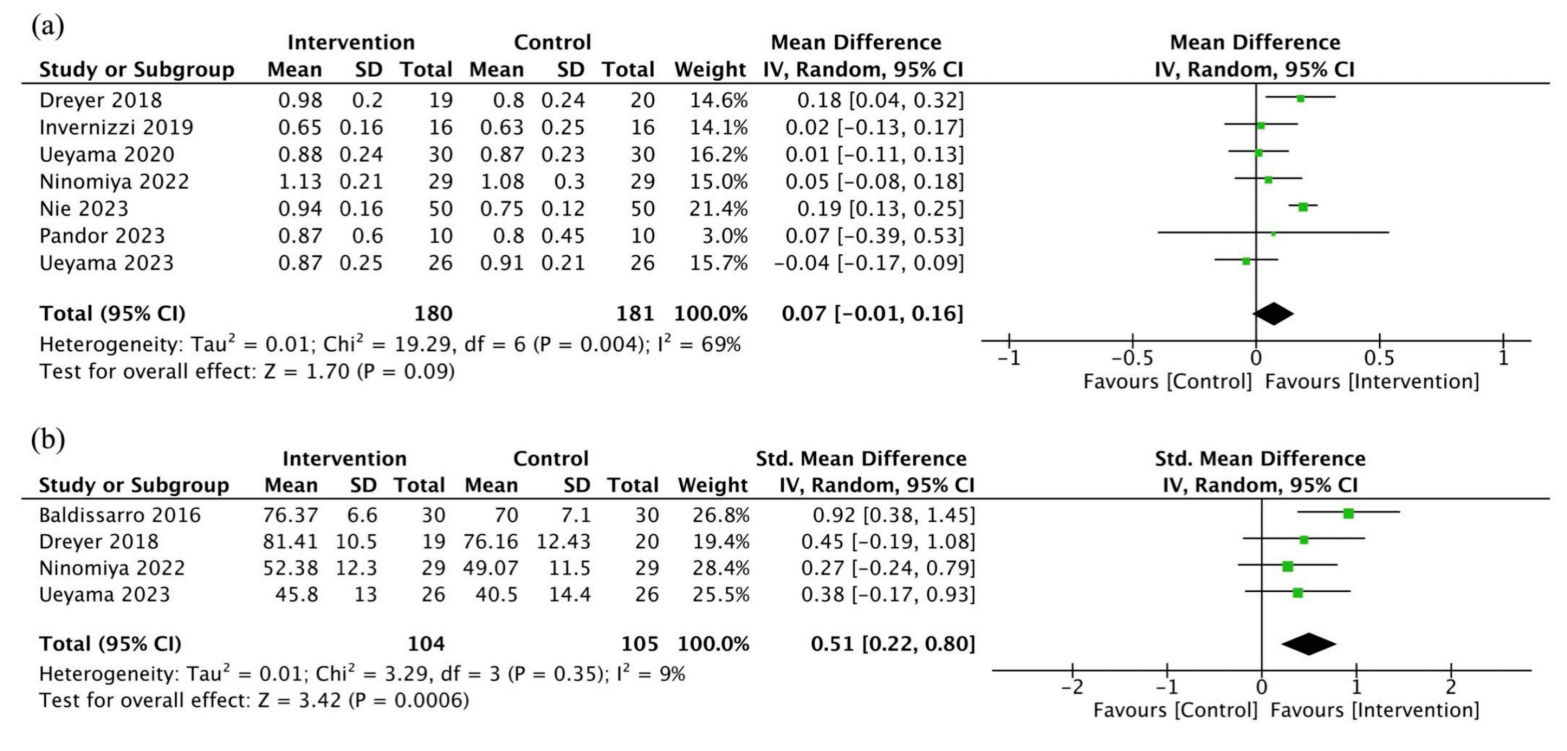
Forest plot of the primary outcomes: (a) Mobility and (b) PRO PRO, patient-reported outcomes

Seven studies (361 patients) reported mobility [10,12,36,38-41]. The studies included patients undergoing TKA (four studies), THA (one study), and hip fracture surgery (two studies). In five of these studies, gait time for four or six meters was measured; thus we calculated gait speed (m/s). In the remaining two studies, TUG was measured. Thus, we converted the results to gait speed based on the previous study [25]. Protein and/or EAA may have resulted in little to no difference in mobility (MD 0.07 m/s, 95% CI: -0.01 to 0.16, I^2^ = 69%, low certainty of evidence) (Figure 3a).

PRO

Four studies (209 patients) reported PRO [10,12,35,36]. The studies included patients undergoing TKA and THA, with two studies for each. Each of these studies used different scales, specifically the KOOS, Japanese Orthopaedic Association Hip-Disease Evaluation Questionnaire (JHEQ), Knee Society Score (KSS), and HHS. Protein and/or EAA may have slightly improved PRO (SMD 0.51, 95% CI: 0.22 to 0.80, I^2^ = 9%, low certainty of evidence) (Figure 3b).

AKI

Seven studies (354 patients) reported no adverse events in both the protein and/or EAA supplementation group and the control group [10,12,33,34,36,37,39]. The studies included patients undergoing TKA (four studies), THA (two studies), and hip fracture surgery (one study). Protein and/or EAA may not have caused AKI with low certainty of evidence.

Secondary Outcomes

**Figure 4:**
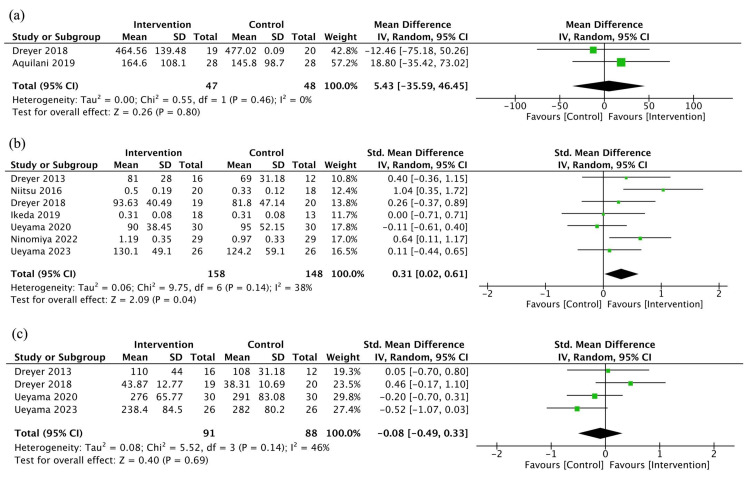
Forest plot of the secondary outcomes: (a) Exercise capacity, (b) muscle strength, and (c) muscle mass

Exercise capacity:* *Two studies (95 patients) reported exercise capacity measured by 6MWT [11,36]. The studies included patients undergoing TKA and hip fracture surgery, with one study for each. The evidence was very uncertain about the effect of protein and/or EAA on exercise capacity (MD 5.43 m, 95% CI: -35.59 to 46.45, I^2^ = 0%, very low certainty of evidence) (Figure 4a).

Muscle strength: Seven studies (306 patients) reported muscle strength [10,12,33,34,36,37,39]. The studies included patients undergoing TKA (four studies), THA (two studies), and hip fracture surgery (one study). Muscle strength was measured using a hand-held dynamometer in four studies, an isokinetic dynamometer in two studies, and the method was unspecified in one study. Protein and/or EAA may have increased muscle strength, but the evidence was very uncertain (SMD 0.31, 95% CI: 0.02 to 0.61, I^2^ = 38%, very low certainty of evidence) (Figure 4b).

Muscle mass*: *Four studies (179 patients) reported muscle mass [10,33,36,39]. All included studies involved patients undergoing TKA. Muscle mass was measured using MRI in two studies to assess volume, and US was used in another two studies to measure cross-sectional area. The evidence was very uncertain about the effect of protein and/or EAA on muscle mass (SMD -0.08, 95% CI: -0.49 to 0.33, I^2 ^= 46%, very low certainty of evidence) (Figure 4c).

All Adverse Events

Seven studies (354 patients) reported no adverse events in both the protein and/or EAA supplementation group and the control group [10,12,33,34,36,37,39]. The studies included patients undergoing TKA (four studies), THA (two studies), and hip fracture surgery (one study). Protein and/or EAA may not have caused adverse events with low certainty of evidence.

Additional Analysis

**Figure 5:**
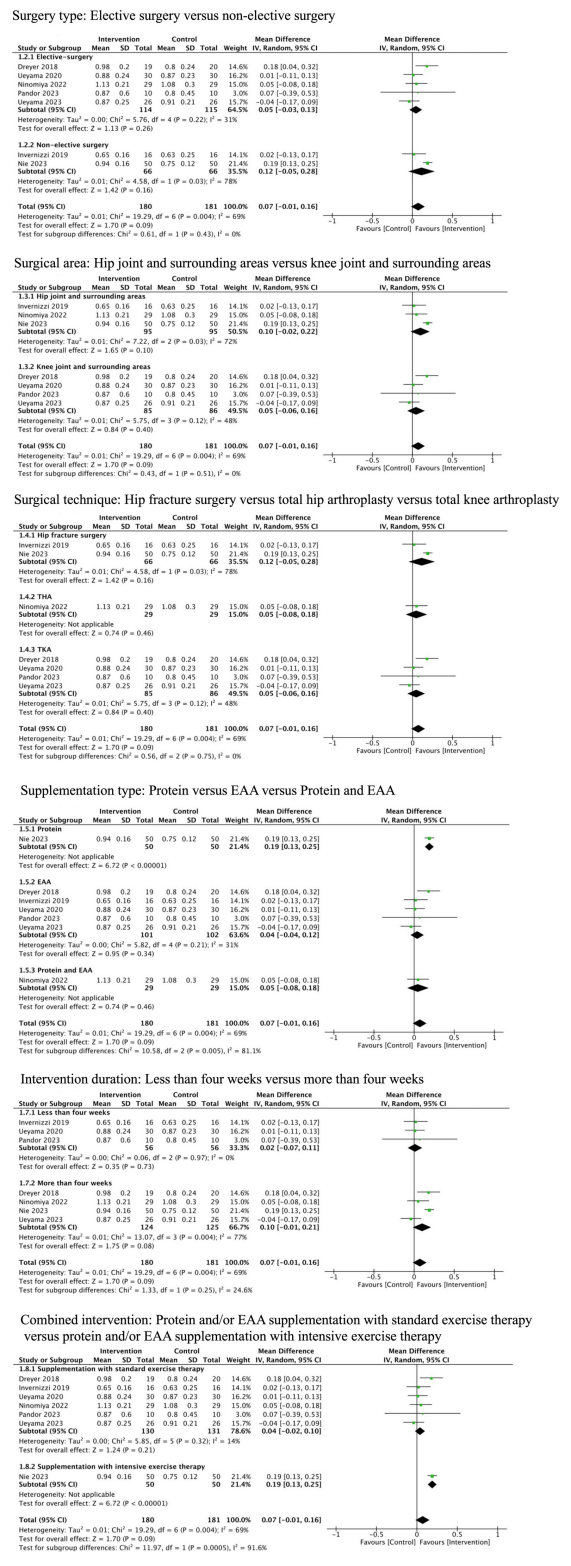
Subgroup analysis for mobility EAA, essential amino acid

We performed prespecified subgroup analyses of the primary and secondary outcomes. Since no studies met the prespecified criteria to form subgroups for some outcomes, we could not conduct some subgroup analyses as planned in the protocol. Regarding supplementation type, there were significant differences in the effects on mobility among the supplementation types (protein: MD 0.19 m/s, 95% CI: 0.13 to 0.25; EAA: MD 0.04 m/s, 95% CI: -0.04 to 0.12; protein and EAA: MD 0.05 m/s, 95% CI: -0.08 to 0.18, p = 0.005 for the interaction) (Figure 5).

**Figure 6:**
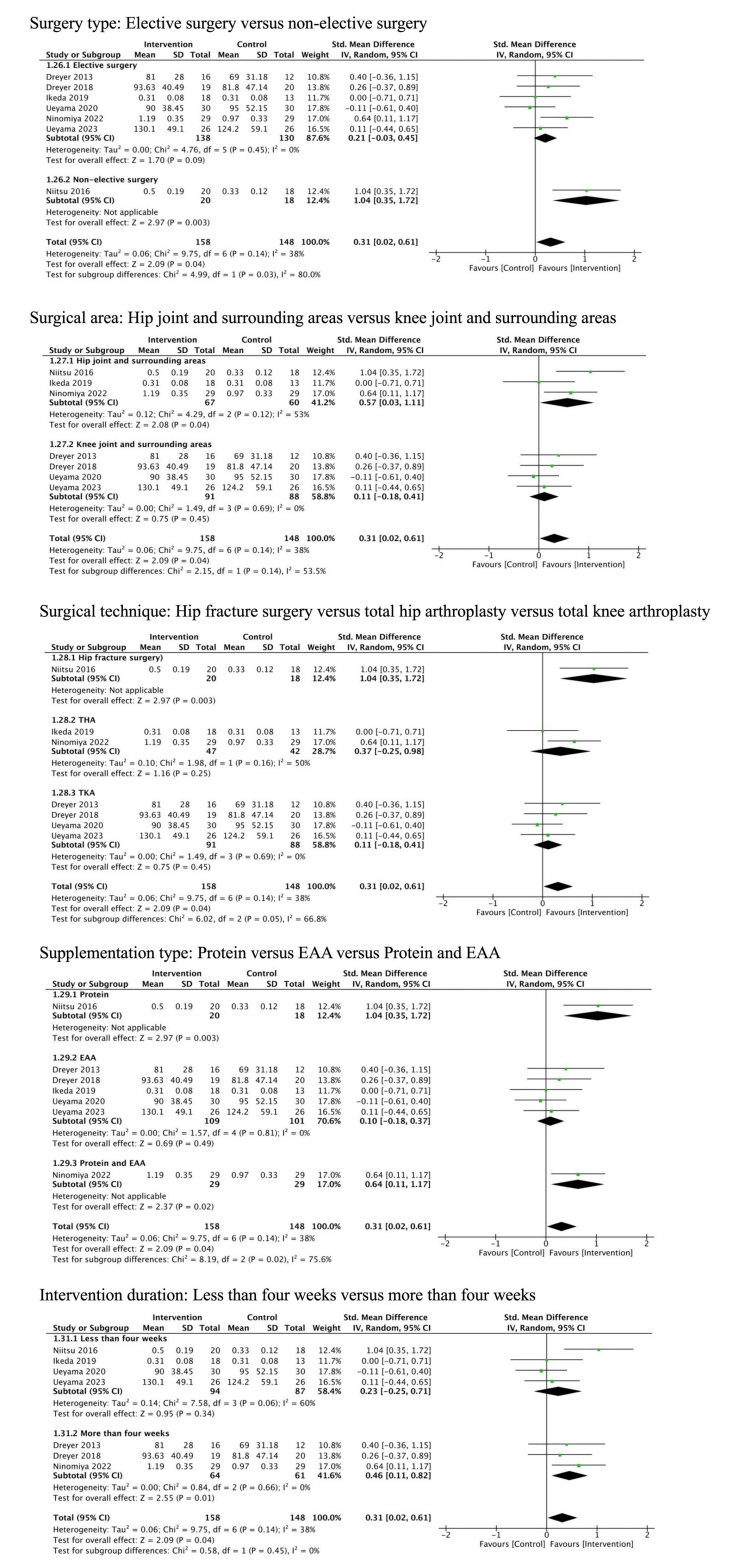
Subgroup analysis for muscle strength EAA, essential amino acid

Furthermore, there were significant differences in the effects on muscle strength among the supplementation types (protein: SMD 1.04, 95%CI: 0.35 to 1.72; EAA: SMD 0.10, 95% CI: -0.18 to 0.37; protein and EAA: SMD 0.64, 95% CI 0.11 to 1.17, p = 0.02 for the interaction) (Figure 6).

Regarding exercise intensity, there was a significant difference in the effects on mobility between supplementation with standard exercise therapy and supplementation with intensive exercise therapy (supplementation with standard exercise therapy: MD 0.04 m/s, 95% CI: -0.02 to 0.10; supplementation with intensive exercise therapy: MD 0.19 m/s, 95% CI: 0.13 to 0.25, p = 0.0005 for the interaction) (Figure 5). Furthermore, regarding surgery type, there was a significant difference in the effects on muscle strength between elective surgeries and non-elective surgeries (elective surgeries: SMD 0.21, 95% CI: -0.03 to 0.45; non-elective surgeries: SMD 1.04, 95% CI: 0.35 to 1.72, p = 0.03 for the interaction) (Figure 6). Other subgroup analyses are presented in Appendix Figures 9-11.

**Figure 7:**
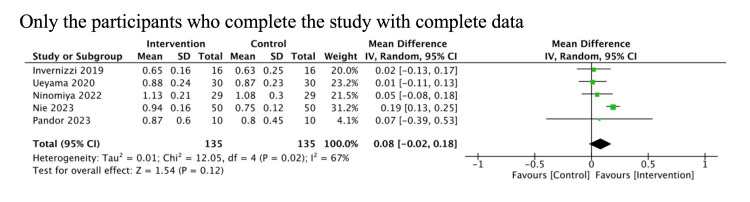
Sensitivity analysis for mobility

**Figure 8:**
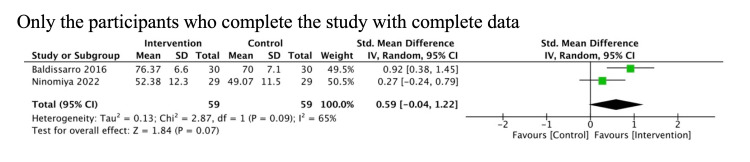
Sensitivity analysis for PRO PRO, patient-reported outcomes

We conducted prespecified sensitivity analyses only for patients who completed the study and provided complete data. The results were similar to the primary analysis (Figures 7, 8).

Other prespecified sensitivity analyses, excluding studies using imputed statistics and including only those defining AKI by clinical guidelines, could not be performed because no appropriate studies were available.

Discussion

This review showed that protein and/or EAA supplementation might slightly improve PRO following lower limb surgery, with low certainty of the evidence. Additionally, it indicated a potential increase in muscle strength, though the certainty of this evidence was very low. There were no reports of AKI or other adverse events. Conversely, it showed no differences in mobility, exercise capacity, and muscle mass with low or very low certainty of the evidence. 

*PRO* 

This review suggested the potential benefits of protein and/or EAA supplementation in improving PRO and muscle strength, extending previous findings to include hip fracture surgeries [14]. The improved PRO (SMD 0.51) indicates a moderate effect according to Cohen's interpretation [42]. The PRO measures in this review comprise various aspects, including physical function, symptoms, activities of daily living, and QOL, with each study using different PRO scales. Improvements may be related to muscle endurance recovery from supplementation, as suggested by previous studies [43,44]. Patients after lower limb surgery typically experience decreased muscle endurance, potentially affecting PRO. Protein and/or EAA supplementation enhances muscle protein synthesis and is purported to accelerate the recovery of muscle endurance [43-45]. Moreover, muscle strength is associated with PRO [46,47]. Protein and/or EAA supplementation might effectively manage these symptoms, positively affecting PRO. Because PRO is directly reported by the patient and reflects components important to the patient, its improvement is valuable [48]. 

Muscle Strength

Our findings suggest that protein and/or EAA supplementation might improve muscle strength across various lower limb surgeries, though the certainty of this evidence is very low. The potential for improving muscle strength with protein and/or EAA supplementation was consistent with previous SRs [14,17]. Surgical stress and immobilization, which promote catabolism or decreased anabolism, often lead to a prolonged decrease in muscle strength following lower limb surgery [2,48-50]. Protein and/or EAA supplementation has been shown to stimulate muscle protein anabolism, suggesting a possibility to improve muscle strength [51]. 

Mobility and Exercise Capacity

Protein and/or EAA supplementation might not improve mobility and exercise capacity. The gait performance recovery including gait speed and exercise capacity is reported to be influenced by various factors. For instance, gait speed recovery after TKA relates to quadriceps muscle strength, knee flexion range of motion, and psychological factors [49]. Similarly, exercise capacity recovery after THA is influenced by several factors, including age, sex, preoperative exercise capacity, and hip range of motion [50]. Therefore, while protein and/or EAA supplementation could improve mobility and exercise capacity through the muscle effects, its overall impact may be limited due to these additional factors. 

Muscle Mass

Muscle mass might not improve with protein and/or EAA supplementation, in contrast to muscle strength. In previous SRs focusing on older adults, the effects of protein and/or EAA supplementation on muscle mass have yet to reach a consensus, with results showing various effects [14,51,52]. Nevertheless, it is essential to note that the studies on muscle strength and mass involved different surgeries. Specifically, all studies on muscle mass involved TKA, which requires invasive quadriceps and knee joint approaches. In contrast, the studies on muscle strength included THA, hip fracture surgery, and TKA. Quadriceps atrophy, common and persistent after TKA and hip surgery, may differ in extent and mechanism due to surgical impact on the quadriceps [33,53]. These differences could affect the efficacy of supplementation on muscle mass and strength, warranting cautious interpretation of the results. Future research should measure muscle mass in THA and hip fracture surgery to identify the effects of protein and/or EAA supplementation for each surgical approach. 

AKI and All Adverse Events

Protein and/or EAA supplementation appeared unlikely to cause any adverse events, including AKI. Previous studies on older adults and patients with chronic heart failure reported no adverse events [14,54,55]. Additionally, another SR suggested that higher protein intake does not worsen kidney function in individuals with normal kidney function, regardless of age or type 2 diabetes [56]. However, most studies in this SR excluded patients with kidney dysfunction or disease. Given the uncertainty about the safety of protein and/or EAA supplementation in individuals with kidney dysfunction or disease, caution might be needed when considering its use in such patients [54,57,58].

Additional Analysis

The subgroup analysis results suggested that supplementation type, exercise intensity, and surgery type might be effect modifiers, influencing several outcomes. Regarding supplementation type, the reports vary with no clear superiority among them [59-61]. Additionally, the impact of exercise intensity on mobility might be influenced by exercise volume rather than exercise load. In studies categorized supplementation with intensive exercise therapy, the control group patients received primarily routine care, such as primary and symptomatic care. In contrast, the intervention group's exercise load was standard and not high-intensity. Previous studies reported that exercise-induced muscle protein synthesis might not necessarily be load-dependent but may be determined by exercise volume [62,63]. Therefore, the impact of exercise load differences on supplementation outcomes remains unclear. Moreover, differential effects of supplementation on muscle strength between elective and non-elective surgeries might be influenced by patient age. Notably, all non-elective surgeries were hip fracture surgeries involving older patients than elective surgery. While muscle protein synthesis induced by protein and/or EAA supplementation has been reported to be age-independent if sufficient amounts are consumed, adaptive responses to exercise are suggested to decline with age [64-66]. Hip fracture patients may also have lower exercise intensity and physical activity than elective surgery patients, possibly due to age. However, the limited number of studies in this SR suggests definitive conclusions on these subgroup analyses cannot be reached. Further research is essential for more conclusive recommendations.

Strength and Limitations

A major strength of this study is that this is the first SR and meta-analysis of RCTs to determine the efficacy and safety of protein and/or EAA supplementation in all lower limb surgery. Furthermore, we used a rigorous methodology following the Cochrane Handbook and GRADE approach and selecting outcomes with established consensus in previous research [18,22-24]. Additionally, this study included the largest number of recently published RCTs, indicating that our findings reflect the latest research developments.

However, this study has several limitations. First, it is essential to note that the studies on each outcome involved different surgeries. Although we conducted subgroup analyses to investigate the differences in effects between surgeries, the limited number of studies in each subgroup requires caution in interpreting these results. Further research is needed to make more conclusive guidance. Second, the risk of bias in all included studies was some concern or high, mainly due to most studies lacking preregistered protocols or predefined analysis plans. Future studies with a low risk of bias are needed to confirm our findings. Third, included RCTs measured outcomes from four weeks to six months after surgery, not clarifying the long-term effects of protein and/or EAA supplementation. Further investigation into the long-term effects of protein and/or EAA supplementation is warranted. Fourth, the effects of protein on muscle mass and physical function are reported to vary by race, suggesting caution when considering the external validity of our findings, especially given the geographic concentration of data in the USA, Italy, Japan, and India [67]. Fifth, caution is needed when extrapolating these findings to younger populations, as the metabolic responses and protein needs can differ significantly between age groups [68]. Since most of the studies in our review focused on middle-aged to older adults, their physiological responses to protein and EAA supplementation may not apply to younger individuals. Lastly, the subgroup and sensitivity analysis were insufficient due to the limited number of studies. Additional studies are necessary to provide more definitive suggestions.

## Conclusions

Our findings suggest that protein and/or EAA supplementation in lower limb surgeries might effectively improve PRO but does not affect mobility. No adverse events associated with supplementation were reported, indicating that this supplementation is generally safe for lower limb surgery patients. However, the low or very low certainty of evidence highlights the need for further well-designed, large-scale RCTs to confirm these findings. Additionally, future research should investigate the differential effects of supplementation across various types of lower limb surgeries to provide more targeted recommendations. Despite these uncertainties, given its low cost and easy accessibility, the medical team and patients might consider protein and/or EAA supplementation as a useful option.

## Figures and Tables

**Figure 9:**
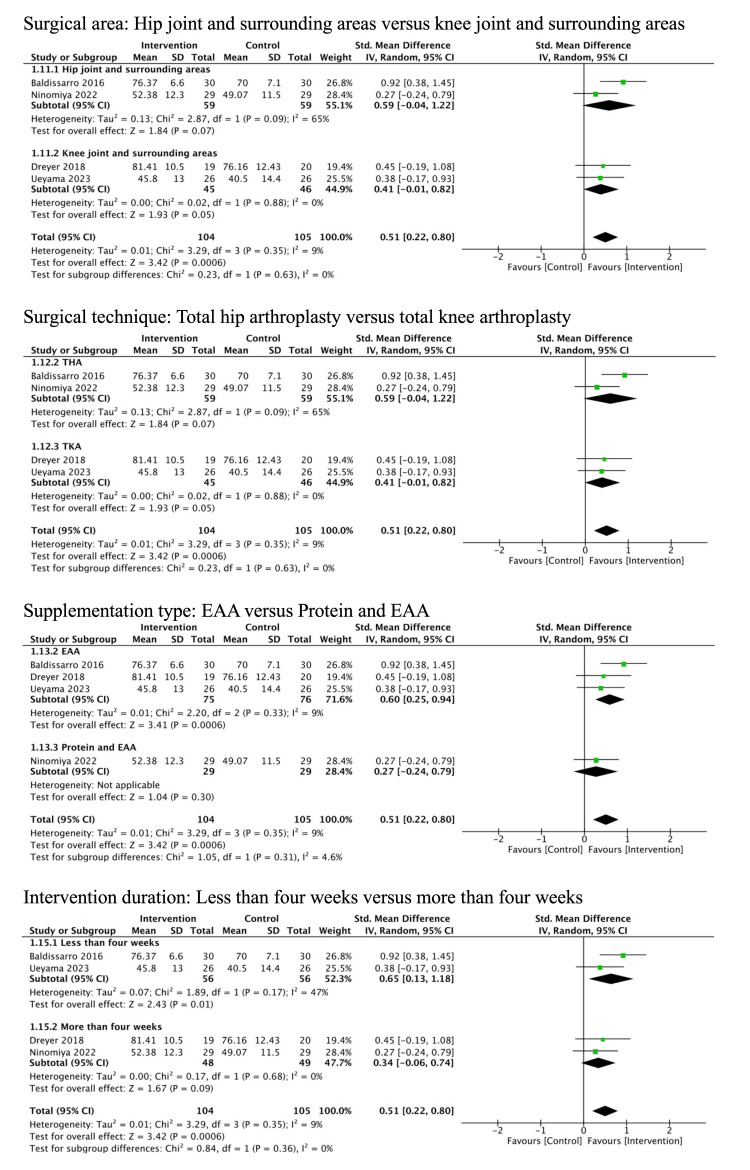
Subgroup analysis for PRO PRO, patient-reported outcomes; EAA, essential amino acid

**Figure 10:**
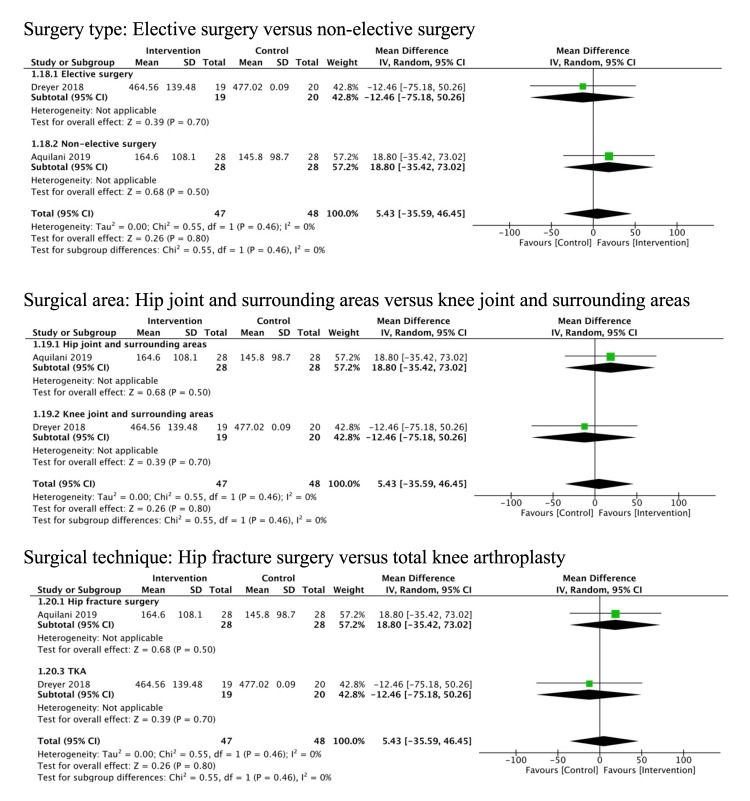
Subgroup analysis for exercise capacity

**Figure 11:**
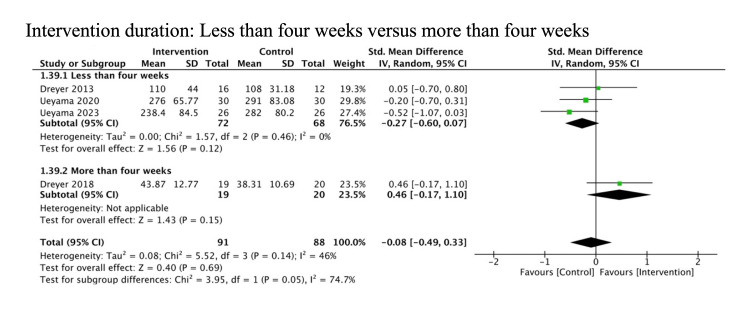
Subgroup analysis for muscle mass

## Appendices

**Table 3:** PRISMA 2020 checklist Source: [19] PRISMA, Preferred Reporting Items for Systematic Reviews and Meta-Analyses

Section and Topic	Item #	Checklist item	Location where item is reported
Title	1	Identify the report as a systematic review.	1
Abstract	2	See the PRISMA 2020 for Abstracts checklist.	Supplementary Tab. S1.
Introduction			
Rationale	3	Describe the rationale for the review in the context of existing knowledge.	3
Objectives	4	Provide an explicit statement of the objective(s) or question(s) the review addresses.	3, 4
Methods			
Eligibility criteria	5	Specify the inclusion and exclusion criteria for the review and how studies were grouped for the syntheses.	4, 5
Information sources	6	Specify all databases, registers, websites, organisations, reference lists and other sources searched or consulted to identify studies. Specify the date when each source was last searched or consulted.	7
Search strategy	7	Present the full search strategies for all databases, registers and websites, including any filters and limits used.	Supplementary Tab. S2.
Selection process	8	Specify the methods used to decide whether a study met the inclusion criteria of the review, including how many reviewers screened each record and each report retrieved, whether they worked independently, and if applicable, details of automation tools used in the process.	7, 8
Data collection process	9	Specify the methods used to collect data from reports, including how many reviewers collected data from each report, whether they worked independently, any processes for obtaining or confirming data from study investigators, and if applicable, details of automation tools used in the process.	6, 7, 8
Data items	10a	List and define all outcomes for which data were sought. Specify whether all results that were compatible with each outcome domain in each study were sought (e.g. for all measures, time points, analyses), and if not, the methods used to decide which results to collect.	6, 7
	10b	List and define all other variables for which data were sought (e.g. participant and intervention characteristics, funding sources). Describe any assumptions made about any missing or unclear information.	6, 7, 8
Study risk of bias assessment	11	Specify the methods used to assess risk of bias in the included studies, including details of the tool(s) used, how many reviewers assessed each study and whether they worked independently, and if applicable, details of automation tools used in the process.	8
Effect measures	12	Specify for each outcome the effect measure(s) (e.g. risk ratio, mean difference) used in the synthesis or presentation of results.	8, 9
Synthesis methods	13a	Describe the processes used to decide which studies were eligible for each synthesis (e.g. tabulating the study intervention characteristics and comparing against the planned groups for each synthesis (item #5)).	8, 9
	13b	Describe any methods required to prepare the data for presentation or synthesis, such as handling of missing summary statistics, or data conversions.	8, 9
	13c	Describe any methods used to tabulate or visually display results of individual studies and syntheses.	9
	13d	Describe any methods used to synthesize results and provide a rationale for the choice(s). If meta-analysis was performed, describe the model(s), method(s) to identify the presence and extent of statistical heterogeneity, and software package(s) used.	8, 9
	13e	Describe any methods used to explore possible causes of heterogeneity among study results (e.g. subgroup analysis, meta-regression).	9
	13f	Describe any sensitivity analyses conducted to assess robustness of the synthesized results.	10
Reporting bias assessment	14	Describe any methods used to assess risk of bias due to missing results in a synthesis (arising from reporting biases).	9
Certainty assessment	15	Describe any methods used to assess certainty (or confidence) in the body of evidence for an outcome.	10, 11
Results			
Study selection	16a	Describe the results of the search and selection process, from the number of records identified in the search to the number of studies included in the review, ideally using a flow diagram.	11, 12, Fig. 1
	16b	Cite studies that might appear to meet the inclusion criteria, but which were excluded, and explain why they were excluded.	Fig. 1
Study characteristics	17	Cite each included study and present its characteristics.	13, 14, Tab. 1
Risk of bias in studies	18	Present assessments of risk of bias for each included study.	11, Supplementary Fig. S1
Results of individual studies	19	For all outcomes, present, for each study: (a) summary statistics for each group (where appropriate) and (b) an effect estimate and its precision (e.g. confidence/credible interval), ideally using structured tables or plots.	12, 13, 14
Results of syntheses	20a	For each synthesis, briefly summarise the characteristics and risk of bias among contributing studies.	12, 13, 14
	20b	Present results of all statistical syntheses conducted. If meta-analysis was done, present for each the summary estimate and its precision (e.g. confidence/credible interval) and measures of statistical heterogeneity. If comparing groups, describe the direction of the effect.	12, 13, 14, Fig. 2, 3,
	20c	Present results of all investigations of possible causes of heterogeneity among study results.	12, 13, 14
	20d	Present results of all sensitivity analyses conducted to assess the robustness of the synthesized results.	14, 15
Reporting biases	21	Present assessments of risk of bias due to missing results (arising from reporting biases) for each synthesis assessed.	NA
Certainty of evidence	22	Present assessments of certainty (or confidence) in the body of evidence for each outcome assessed.	Tab. 2
Discussion	23a	Provide a general interpretation of the results in the context of other evidence.	16, 17, 18, 19
	23b	Discuss any limitations of the evidence included in the review.	20
	23c	Discuss any limitations of the review processes used.	20
	23d	Discuss implications of the results for practice, policy, and future research.	21
Other Information			
Registration and protocol	24a	Provide registration information for the review, including register name and registration number, or state that the review was not registered.	4
	24b	Indicate where the review protocol can be accessed, or state that a protocol was not prepared.	4
	24c	Describe and explain any amendments to information provided at registration or in the protocol.	11
Support	25	Describe sources of financial or non-financial support for the review, and the role of the funders or sponsors in the review.	21
Competing interests	26	Declare any competing interests of review authors.	21
Availability of data, code and other materials	27	Report which of the following are publicly available and where they can be found: template data collection forms; data extracted from included studies; data used for all analyses; analytic code; any other materials used in the review.	Supplementary Tab. S2, 3

Section and Topic	Item #	Checklist item	Reported (Yes/No)
TITLE			
Title	1	Identify the report as a systematic review.	Yes
Background			
Objectives	2	Provide an explicit statement of the main objective(s) or question(s) the review addresses.	Yes
Methods			
Eligibility criteria	3	Specify the inclusion and exclusion criteria for the review.	Yes
Information sources	4	Specify the information sources (e.g. databases, registers) used to identify studies and the date when each was last searched.	Yes
Risk of bias	5	Specify the methods used to assess risk of bias in the included studies.	Yes
Synthesis of results	6	Specify the methods used to present and synthesise results.	Yes
Results			
Included studies	7	Give the total number of included studies and participants and summarise relevant characteristics of studies.	Yes
Synthesis of results	8	Present results for main outcomes, preferably indicating the number of included studies and participants for each. If meta-analysis was done, report the summary estimate and confidence/credible interval. If comparing groups, indicate the direction of the effect (i.e. which group is favoured).	Yes
Discussion			
Limitations of evidence	9	Provide a brief summary of the limitations of the evidence included in the review (e.g. study risk of bias, inconsistency and imprecision).	Yes
Interpretation	10	Provide a general interpretation of the results and important implications.	Yes
Other			
Funding	11	Specify the primary source of funding for the review.	NA
Registration	12	Provide the register name and registration number.	NA

**Table 4:** Search strategy

MEDLINE (PubMed) search strategy
#1 “Lower Extremity/injuries”[mh]
#2 “Lower Extremity/surgery”[mh]
#3 “Lower limb*”[tiab]
#4 “Lower extremity*”[tiab]
#5 “Fracture Fixation“[mh]
#6 “Fracture Fixation*“[tiab]
#7 “Hip Fractures”[mh]
#8 “Arthroplasty, Replacement, Hip”[mh]
#9 “Hip Fractures*”[tiab]
#10 “Femoral Fracture*”[tiab]
#11 “Hip injur*”[tiab]
#12 “total hip arthroplasty*”[tiab]
#13 “total hip replacement*”[tiab]
#14 “Arthroplasty, Replacement, Knee”[mh]
#15 “Anterior Cruciate Ligament Reconstruction”[mh]
#16 “Anterior Cruciate Ligament“[mh]
#17 “Anterior Cruciate Ligament Injuries”[mh]
#18 “Knee Injur*”[tiab]
#19 “total knee arthroplasty*”[tiab]
#20 “total knee replacement*”[tiab]
#21 “Anterior Cruciate Ligament*”[tiab]
#22 “Ankle Injuries”[mh]
#23 “Ankle Fractures”[mh]
#24 “Achilles Tendon”[mh]
#25 “Arthroplasty, Replacement, Ankle”[mh]
#26 “Ankle injur*”[tiab]
#27 “total ankle arthroplasty*”[tiab]
#28 “total ankle replacement*”[tiab]
#29 “Achilles Tendon*”[tiab]
#30 #1 OR #2 OR #3 OR #4 OR #5 OR #6 OR #7 OR #8 OR #9 OR #10 OR #11 OR #12 OR #13 OR #14 OR #15 OR #16 OR #17 OR #18 OR #19 OR #20 OR #21 OR #22 OR #23 OR #24 OR #25 OR #26 OR #27 OR #28 OR #29
#31 “Nutrition Therapy”[mh]
#32 “Nutrition Therapy”[tiab]
#33 “Dietary Supplements”[mh]
#34 “Dietary Proteins”[mh]
#35 “Diet, High-Protein”[mh]
#36 “Amino Acids“[mh]
#37 “Amino Acids“[tiab]
#38 “Amino Acids, Essential”[mh]
#39 “Amino Acids, Branched-Chain”[mh]
#40 Leucine[mh]
#41 “Essential Amino Acid*”[tiab]
#42 “Branched-chain amino acid*”[tiab]
#43 Protein*[tiab]
#44 Supplement*[tiab]
#45 Leucine[tiab]
#46 #31 OR #32 OR #33 OR #34 OR #35 OR #36 OR #37 OR #38 OR #39 OR #40 OR #41 OR #42 OR #43 OR #44 OR #45
#47 #30 AND #46
#48 "randomized controlled trial"[pt]
#49 "controlled clinical trial"[pt]
#50 "randomized"[tiab]
#51"randomly" [tiab]
#52 "trial"[tiab]
#53 #48 OR #49 OR #50 OR #51 OR #52
#54 #47 AND #53

CENTRAL (Cochrane Library) search strategy
([mh "Lower Extremity"] OR ("Lower" NEXT limb):ti,ab OR ("Lower" NEXT extremity):ti,ab OR [mh "Fracture Fixation"] OR ("Fracture" NEXT Fixation):ti,ab OR [mh "Hip Fractures"] OR [mh "Arthroplasty, Hip Replacement"] OR ("Hip" NEXT Fractures):ti,ab OR ("Femoral" NEXT Fracture):ti,ab OR ("Hip" NEXT injury):ti,ab OR ("total hip" NEXT arthroplasty):ti,ab OR ("total hip" NEXT replacement):ti,ab OR [mh "Arthroplasty, Knee"] OR [mh "Arthroplasty, Knee Replacement"] OR [mh "ACL Injuries"] OR [mh "ACL Injury"] OR ("Knee" NEXT Injury):ti,ab OR ("total knee" NEXT arthroplasty):ti,ab OR ("total knee" NEXT replacement):ti,ab OR ("Anterior Cruciate" NEXT Ligament):ti,ab OR [mh "Ankle Injuries"] OR [mh "Ankle Fractures"] OR [mh "Achilles Tendon"] OR [mh "Arthroplasty, Replacement, Ankle"] OR ("Ankle" NEXT injury):ti,ab OR ("total ankle" NEXT arthroplasty):ti,ab OR ("total ankle" NEXT replacement):ti,ab OR ("Achilles" NEXT Tendon):ti,ab) AND ("​​Nutrition Therapy":ti,ab OR [mh "Dietary Supplements"] OR [mh "Dietary Proteins"] OR [mh "Diet, High-Protein"] OR [mh "Amino Acids"] OR "Amino Acids":ti,ab OR [mh "Amino Acids, Essential"] OR [mh "Amino Acids, Branched Chain"] OR [mh Leucine] OR ("Essential Amino" NEXT Acid*):ti,ab OR ("Branched-chain amino" NEXT acid*):ti,ab OR Protein*:ti,ab OR Supplement:ti,ab OR Leucine:ti,ab)

EMBASE (Dialog) search strategy
EMB.EXACT.EXPLODE("leg injury")
TI("Lower limb*") OR AB("Lower limb*")
TI("Lower extremity*") OR AB("Lower extremity*")
EMB.EXACT.EXPLODE("Fracture Fixation")
TI("Fracture Fixation*") OR AB("Fracture Fixation*")
EMB.EXACT.EXPLODE("Hip Fractures")
EMB.EXACT.EXPLODE("hip replacement")
TI("Hip Fractures*") OR AB("Hip Fractures*")
TI("Femoral Fracture*") OR AB("Femoral Fracture*")
TI("Hip injur*") OR AB("Hip injur*")
TI("total hip arthroplasty*") OR AB("total hip arthroplasty*")
TI("total hip replacement*") OR AB("total hip replacement*")
EMB.EXACT.EXPLODE("knee replacement")
EMB.EXACT.EXPLODE("Anterior Cruciate Ligament Reconstruction")
EMB.EXACT.EXPLODE("Anterior Cruciate Ligament")
EMB.EXACT.EXPLODE("Anterior Cruciate Ligament Injury")
TI("Knee Injur*") OR AB("Knee Injur*")
TI("total knee arthroplasty*") OR AB("total knee arthroplasty*")
TI("total knee replacement*") OR AB("total knee replacement*")
TI("Anterior Cruciate Ligament*") OR AB("Anterior Cruciate Ligament*")
EMB.EXACT.EXPLODE("Ankle Injuries")
EMB.EXACT.EXPLODE("Ankle Fracture")
EMB.EXACT.EXPLODE("Achilles Tendon")
EMB.EXACT.EXPLODE("ankle replacement")
TI("Ankle injur*") OR AB("Ankle injur*")
TI("total ankle arthroplasty*") OR AB("total ankle arthroplasty*")
TI("total ankle replacement*") OR AB("total ankle replacement*")
TI("Achilles Tendon*") OR AB("Achilles Tendon*")
S1 OR S2 OR S3 OR S4 OR S5 OR S6 OR S7 OR S8 OR S9 OR S10 OR S11 OR S12 OR S13 OR S14 OR S15 OR S16 OR S17 OR S18 OR S19 OR S20 OR S21 OR S22 OR S23 OR S24 OR S25 OR S26 OR S27 OR S28
EMB.EXACT.EXPLODE("diet therapy")
TI("Nutrition Therapy") OR AB("Nutrition Therapy")
EMB.EXACT.EXPLODE("Dietary Supplement")
EMB.EXACT.EXPLODE("protein intake")
EMB.EXACT.EXPLODE("protein diet")
EMB.EXACT.EXPLODE("Amino Acid")
TI("Amino Acids") OR AB("Amino Acids")
EMB.EXACT.EXPLODE("essential amino acid")
EMB.EXACT.EXPLODE("branched chain amino acid")
EMB.EXACT.EXPLODE("Leucine")
TI("Essential Amino Acid*") OR AB("Essential Amino Acid*")
TI("Branched-chain amino acid*") OR AB("Branched-chain amino acid*")
TI(Protein*) OR AB(Protein*)
TI(Supplement*) OR AB(Supplement*)
TI(Leucine) OR AB(Leucine)
S30 OR S31 OR S32 OR S33 OR S34 OR S35 OR S36 OR S37 OR S38 OR S39 OR S40 OR S41 OR S42 OR S43 OR S44
S29 AND S45
(ab(random*) OR ti(random*)) OR (ab(placebo*) OR ti(placebo*)) OR (ab(double NEAR/1 blind*) OR ti(double NEAR/1 blind*))
S46 AND S47

ICTRP search strategy
("Lower Extremity surger*" OR "Lower limb surger*" OR “Fracture Fixation” OR “Hip Fracture*” OR “Femoral Fracture” OR “Hip injur*” OR “total hip arthroplasty” OR “total hip replacement” OR “total knee arthroplasty” OR “total knee replacement” OR “Anterior Cruciate Ligament*“ OR “Knee Injur*” OR “Ankle Injur*” OR “Ankle Fracture*” OR “total ankle arthroplasty” OR “total ankle replacement” OR “Achilles Tendon*”)
AND
("Dietary Supplements" OR “Dietary Proteins” OR “Essential Amino Acid*” OR “Branched chain amino acid*” OR “Leucine” OR “Protein*” OR “Supplement*”)

ClinicalTrials.gov search strategy
Condition or disease:
("Lower Extremity surgery" OR "Lower limb surgery" OR “Fracture Fixation” OR “Hip Fracture” OR “Femoral Fracture” OR “Hip injury” OR “total hip arthroplasty” OR “total hip replacement” OR “total knee arthroplasty” OR “total knee replacement” OR “Anterior Cruciate Ligament“ OR “Knee Injury” OR “Ankle Injury” OR “Ankle Fracture” OR “total ankle arthroplasty” OR “total ankle replacement” OR “Achilles Tendon”)
Intervention:
("Dietary Supplements" OR “Dietary Proteins” OR “Essential Amino Acid” OR “Branched chain amino acid” OR Leucine OR Protein OR Supplement OR Supplementation)

**Table 5:** Excluded studies and reasons for exclusion

Reason for exclusion	Title
Wrong intervention	Anbar R, Beloosesky Y, Cohen J, et al. Tight calorie control in geriatric patients following hip fracture decreases complications: a randomized, controlled study. Clin Nutr. 2014;33(1):23-28. doi:10.1016/j.clnu.2013.03.005
Wrong outcome	Aquilani R, Zuccarelli GC, Condino AM, et al. Despite Inflammation, Supplemented Essential Amino Acids May Improve Circulating Levels of Albumin and Haemoglobin in Patients after Hip Fractures. Nutrients. 2017;9(6):637. Published 2017 Jun 21. doi:10.3390/nu9060637
Wrong study design	Askanazi J, Furst P, Michelsen CB, et al. Muscle and plasma amino acids after injury: hypocaloric glucose vs. amino acid infusion. Ann Surg. 1980;191(4):465-472. doi:10.1097/00000658-198004000-00013
Wrong study design	Avenell A, Smith TO, Curtain JP, Mak JC, Myint PK. Nutritional supplementation for hip fracture aftercare in older people. Cochrane Database Syst Rev. 2016;11(11):CD001880. Published 2016 Nov 30. doi:10.1002/14651858.CD001880.pub6
Wrong intervention	Bachrach-Lindström M, Johansson T, Unosson M, Ek AC, Wahlström O. Nutritional status and functional capacity after femoral neck fractures: a prospective randomized one-year follow-up study. Aging (Milano). 2000;12(5):366-374. doi:10.1007/BF03339862
Wrong study design	Bailey AN, Hocker AD, Vermillion BR, et al. MAFbx, MuRF1, and the stress-activated protein kinases are upregulated in muscle cells during total knee arthroplasty. Am J Physiol Regul Integr Comp Physiol. 2012;303(4):R376-R386. doi:10.1152/ajpregu.00146.2012
Wrong intervention	Bastow MD, Rawlings J, Allison SP. Benefits of supplementary tube feeding after fractured neck of femur: a randomised controlled trial. Br Med J (Clin Res Ed). 1983;287(6405):1589-1592.
Wrong outcome	Botella-Carretero JI, Iglesias B, Balsa JA, Arrieta F, Zamarrón I, Vázquez C. Perioperative oral nutritional supplements in normally or mildly undernourished geriatric patients submitted to surgery for hip fracture: a randomized clinical trial. Clin Nutr. 2010;29(5):574-579. doi:10.1016/j.clnu.2010.01.012
Wrong outcome	Botella-Carretero JI, Iglesias B, Balsa JA, Zamarrón I, Arrieta F, Vázquez C. Effects of oral nutritional supplements in normally nourished or mildly undernourished geriatric patients after surgery for hip fracture: a randomized clinical trial. JPEN J Parenter Enteral Nutr. 2008;32(2):120-128. doi:10.1177/0148607108314760
Wrong intervention	Breedveld-Peters JJ, Reijven PL, Wyers CE, et al. Integrated nutritional intervention in the elderly after hip fracture. A process evaluation. Clin Nutr. 2012;31(2):199-205. doi:10.1016/j.clnu.2011.10.004
Wrong intervention	Breedveld-Peters, José JL, et al. Barriers and facilitators of nutritional intervention after hip fracture in integrated care as perceived by the different health care professionals: a qualitative interview study. e-SPEN Journal 7.5 (2012): e182-e188.
Wrong intervention	Cao G, Huang Q, Xu B, Huang Z, Xie J, Pei F. Multimodal Nutritional Management in Primary Total Knee Arthroplasty: A Randomized Controlled Trial. J Arthroplasty. 2017;32(11):3390-3395. doi:10.1016/j.arth.2017.06.020
Wrong outcome	Carlsson P, Tidermark J, Ponzer S, Söderqvist A, Cederholm T. Food habits and appetite of elderly women at the time of a femoral neck fracture and after nutritional and anabolic support. J Hum Nutr Diet. 2005;18(2):117-120. doi:10.1111/j.1365-277X.2005.00594.x
Wrong outcome	Chevalley T, Hoffmeyer P, Bonjour JP, Rizzoli R. Early serum IGF-I response to oral protein supplements in elderly women with a recent hip fracture. Clin Nutr. 2010;29(1):78-83. doi:10.1016/j.clnu.2009.07.003
Wrong outcome	Church DD, Schutzler SE, Wolfe RR, Ferrando AA. Perioperative amino acid infusion reestablishes muscle net balance during total hip arthroplasty. Physiol Rep. 2021;9(18):e15055. doi:10.14814/phy2.15055
Wrong study design	de Tecnologías Sanitarias, I. D. E. Nutrición enteral oral domiciliaria en pacientes mayores con fractura de cadera y desnutrición.
Wrong outcome	Delmi M, Rapin CH, Bengoa JM, Delmas PD, Vasey H, Bonjour JP. Dietary supplementation in elderly patients with fractured neck of the femur. Lancet. 1990;335(8696):1013-1016. doi:10.1016/0140-6736(90)91073-j
Wrong outcome	Deng Y, Fang Y, Li H, et al. A preoperative whey protein and glucose drink before hip fracture surgery in the aged improves symptomatic and metabolic recovery. Asia Pac J Clin Nutr. 2020;29(2):234-238. doi:10.6133/apjcn.202007_29(2).0004
Wrong intervention	Ekinci O, Yanık S, Terzioğlu Bebitoğlu B, Yılmaz Akyüz E, Dokuyucu A, Erdem Ş. Effect of Calcium β-Hydroxy-β-Methylbutyrate (CaHMB), Vitamin D, and Protein Supplementation on Postoperative Immobilization in Malnourished Older Adult Patients With Hip Fracture: A Randomized Controlled Study. Nutr Clin Pract. 2016;31(6):829-835. doi:10.1177/0884533616629628
Wrong intervention	Eneroth M, Olsson UB, Thorngren KG. Insufficient fluid and energy intake in hospitalised patients with hip fracture. A prospective randomised study of 80 patients. Clin Nutr. 2005;24(2):297-303. doi:10.1016/j.clnu.2004.12.003
Wrong intervention	Eneroth M, Olsson UB, Thorngren KG. Nutritional supplementation decreases hip fracture-related complications. Clin Orthop Relat Res. 2006;451:212-217. doi:10.1097/01.blo.0000224054.86625.06
Wrong outcome	Espaulella J, Guyer H, Diaz-Escriu F, Mellado-Navas JA, Castells M, Pladevall M. Nutritional supplementation of elderly hip fracture patients. A randomized, double-blind, placebo-controlled trial. Age Ageing. 2000;29(5):425-431. doi:10.1093/ageing/29.5.425
Wrong outcome	Fabian E, Gerstorfer I, Thaler HW, Stundner H, Biswas P, Elmadfa I. Nutritional supplementation affects postoperative oxidative stress and duration of hospitalization in patients with hip fracture. Wien Klin Wochenschr. 2011;123(3-4):88-93. doi:10.1007/s00508-010-1519-6
Wrong outcome	Flodin L, Cederholm T, Sääf M, et al. Effects of protein-rich nutritional supplementation and bisphosphonates on body composition, handgrip strength and health-related quality of life after hip fracture: a 12-month randomized controlled study. BMC Geriatr. 2015;15:149. Published 2015 Nov 17. doi:10.1186/s12877-015-0144-7
Wrong outcome	Flodin L, Sääf M, Cederholm T, et al. Additive effects of nutritional supplementation, together with bisphosphonates, on bone mineral density after hip fracture: a 12-month randomized controlled study. Clin Interv Aging. 2014;9:1043-1050. Published 2014 Jul 7. doi:10.2147/CIA.S63987
Wrong study design	Hamill MJ, Afeyan R, Chakravarthy MV, Tramontin T. Endogenous Metabolic Modulators: Emerging Therapeutic Potential of Amino Acids. iScience. 2020;23(10):101628. Published 2020 Sep 29. doi:10.1016/j.isci.2020.101628
Wrong intervention	Hartgrink HH, Wille J, König P, Hermans J, Breslau PJ. Pressure sores and tube feeding in patients with a fracture of the hip: a randomized clinical trial. Clin Nutr. 1998;17(6):287-292. doi:10.1016/s0261-5614(98)80321-x
Wrong intervention	Hendrickson NR, Davison J, Glass NA, et al. Conditionally Essential Amino Acid Supplementation Reduces Postoperative Complications and Muscle Wasting After Fracture Fixation: A Randomized Controlled Trial. J Bone Joint Surg Am. 2022;104(9):759-766. doi:10.2106/JBJS.21.01014
Wrong intervention	Henrotin, Yves, et al. Pomegranate extract to improve muscle recovery after anterior cruciate ligament plasty? Results of an exploratory, randomized and double-blind study. Tissue Engineering Part a. Vol. 28. 140 HUGUENOT STREET, 3RD FL, New Rochelle, NY 10801 USA: Mart Ann Liebert, INC, 2022.
Wrong outcome	Hermanky, Maria, et al. Effects of a protein optimized diet combined with moderate resistance training on the postoperative course in older patients with hip fracture. AKTUELLE ERNAHRUNGSMEDIZIN 42.3 (2017): 180-187.
Wrong intervention	Hocker AD, Boileau RM, Lantz BA, Jewett BA, Gilbert JS, Dreyer HC. Endoplasmic Reticulum Stress Activation during Total Knee Arthroplasty. Physiol Rep. 2013;1(3):e00052. doi:10.1002/phy2.52
Wrong outcome	Hocker, A.D., Bailey, A.N., Senesac, H.A., Ratchford, S.M., Jewett, B.A., Lantz, B.A., Shah, S.N. and Dreyer, H.C. (2012), Effects of twice-daily ingestion of essential amino acids on amino acid transporter transcript and protein expression in older adults prior to total knee arthroplasty. FASEB J, 26: 1086.8-1086.8.
Wrong population	Holm L, Esmarck B, Mizuno M, et al. The effect of protein and carbohydrate supplementation on strength training outcome of rehabilitation in ACL patients. J Orthop Res. 2006;24(11):2114-2123. doi:10.1002/jor.20147
Wrong intervention	Houwing RH, Rozendaal M, Wouters-Wesseling W, Beulens JW, Buskens E, Haalboom JR. A randomised, double-blind assessment of the effect of nutritional supplementation on the prevention of pressure ulcers in hip-fracture patients. Clin Nutr. 2003;22(4):401-405. doi:10.1016/s0261-5614(03)00039-6
Wrong outcome	Howard EE, Margolis LM, Fussell MA, et al. Effect of High-Protein Diets on Integrated Myofibrillar Protein Synthesis before Anterior Cruciate Ligament Reconstruction: A Randomized Controlled Pilot Study. Nutrients. 2022;14(3):563. Published 2022 Jan 27. doi:10.3390/nu14030563
Wrong study design	Hulsbæk S, Ban I, Aasvang TK, et al. Preliminary effect and feasibility of physiotherapy with strength training and protein-rich nutritional supplement in combination with anabolic steroids in cross-continuum rehabilitation of patients with hip fracture: protocol for a blinded randomized controlled pilot trial (HIP-SAP1 trial). Trials. 2019;20(1):763. Published 2019 Dec 23. doi:10.1186/s13063-019-3845-y
Wrong intervention	Hulsbæk S, Bandholm T, Ban I, et al. Feasibility and preliminary effect of anabolic steroids in addition to strength training and nutritional supplement in rehabilitation of patients with hip fracture: a randomized controlled pilot trial (HIP-SAP1 trial). BMC Geriatr. 2021;21(1):323. Published 2021 May 20. doi:10.1186/s12877-021-02273-z
Wrong study design	Hulsbæk S, Laursen LB, Kristensen MT, Midtgaard J. Older patients' perspectives on participating in multimodal rehabilitation including anabolic steroids following hip fracture: a qualitative study embedded within a pilot RCT. Disabil Rehabil. 2023;45(1):81-89. doi:10.1080/09638288.2022.2025929
Wrong intervention	J.H. Kang, D.W. Shin, H.W. Baik, J. Hong, LB039-MON Short-term oral nutritional supplements and nutrition intervention in elderly patients after hip fracture surgery: a randomized controlled clinical trial, Clinical Nutrition Supplements, Volume 7, Issue 1, 2012, Page 280,
Wrong study design	Jones, L. C., et al. Impact of oral nutritional supplementation on hip fracture outcomes. OSTEOPOROSIS INTERNATIONAL. Vol. 25. 236 GRAYS INN RD, 6TH FLOOR, LONDON WC1X 8HL, ENGLAND: SPRINGER LONDON LTD, 2014.
Wrong study design	Kouw IWK, Groen BBL, Smeets JSJ, et al. One Week of Hospitalization Following Elective Hip Surgery Induces Substantial Muscle Atrophy in Older Patients. J Am Med Dir Assoc. 2019;20(1):35-42. doi:10.1016/j.jamda.2018.06.018
Wrong outcome	Laboute E, France J, Trouve P, Puig PL, Boireau M, Blanchard A. Rehabilitation and leucine supplementation as possible contributors to an athlete's muscle strength in the reathletization phase following anterior cruciate ligament surgery. Ann Phys Rehabil Med. 2013;56(2):102-112. doi:10.1016/j.rehab.2012.11.002
Wrong intervention	Lan, Yuqing, et al. The effect of NRS2002-guided nutrition interventions on health knowledge, nutrition, and fracture union in senior patients with hip fractures after surgery. Int J Clin Exp Med 12.12 (2019): 13455-13463.
Wrong intervention	Liaw KY, Askanazi J, Michelsen CB, Furst PF, Elwyn DH, Kinney JM. Effect of postoperative nutrition on muscle high energy phosphates. Ann Surg. 1982;195(1):12-18. doi:10.1097/00000658-198201001-00002
Wrong intervention	López-Vidriero E, Olivé-Vilas R, López-Capapé D, Varela-Sende L, López-Vidriero R, Til-Pérez L. Efficacy and Tolerability of Progen, a Nutritional Supplement Based on Innovative Plasma Proteins, in ACL Reconstruction: A Multicenter Randomized Controlled Trial. Orthop J Sports Med. 2019;7(2):2325967119827237. Published 2019 Feb 27. doi:10.1177/2325967119827237
Wrong intervention	Luo, M., et al. PP097-MON ORAL NUTRITIONAL SUPPLEMENT (ONS) IMPROVED NUTRITIONAL STATUS IN MALNOURISHED PATIENTS RECEIVING HIP FRACTURE SURGERY. Clinical Nutrition Supplements 1.6 (2011): 151.
Wrong study design	Malafarina V, Uriz-Otano F, Gil-Guerrero L, Iniesta R, Zulet MA, Martinez JA. Study protocol: High-protein nutritional intervention based on β-hydroxy-β-methylbutirate, vitamin D3 and calcium on obese and lean aged patients with hip fractures and sarcopenia. The HIPERPROT-GER study. Maturitas. 2013;76(2):123-128. doi:10.1016/j.maturitas.2013.06.016
Wrong outcome	Mendonça GO, Severino MLB, Oliveira KM, et al. The effects of neuromuscular electrical stimulation in association with whey protein supplementation after anterior cruciate ligament reconstruction. Acta Ortop Bras. 2021;29(6):316-322. doi:10.1590/1413-785220212906237983
Wrong study design	MENDONÇA, Gabriela Otília. Efeitos da suplementação com Whey Protein associada à estimulação elétrica neuromuscular do quadríceps femoral após reconstrução do ligamento cruzado anterior. (2018).
Wrong intervention	Miller MD, Crotty M, Whitehead C, Bannerman E, Daniels LA. Nutritional supplementation and resistance training in nutritionally at risk older adults following lower limb fracture: a randomized controlled trial. Clin Rehabil. 2006;20(4):311-323. doi:10.1191/0269215506cr942oa
Wrong intervention	Miller MD, Foley A, Gunn SM, Crotty M. Progression and adherence to an individually prescribed and supervised resistance training intervention in older adults recovering in hospital from lower limb fragility fracture. Patient Prefer Adherence. 2008;2:107-113. Published 2008 Feb 2.
Wrong intervention	Miller, Aspen, et al. Amino acid supplementation is associated with reduced mortality and complications following acute fracture fixation: results of a prospective, randomized controlled trial 4.Supplement_2 (2020): 1134-1134.
Wrong intervention	Milte R, Miller MD, Crotty M, et al. Cost-effectiveness of individualized nutrition and exercise therapy for rehabilitation following hip fracture. J Rehabil Med. 2016;48(4):378-385. doi:10.2340/16501977-2070
Wrong intervention	Milte, Rachel Kathleen. Economic Evaluation of Multidisciplinary Rehabilitation Following Hip Fracture. Diss. Flinders University, School of Health Sciences., 2014.
Wrong outcome	Muyskens JB, Foote DM, Bigot NJ, et al. Cellular and morphological changes with EAA supplementation before and after total knee arthroplasty. J Appl Physiol (1985). 2019;127(2):531-545. doi:10.1152/japplphysiol.00869.2018
Wrong outcome	Muyskens JB, Winbush A, Foote DM, Turnbull DW, Dreyer HC. Essential amino acid supplementation alters the p53 transcriptional response and cytokine gene expression following total knee arthroplasty. J Appl Physiol (1985). 2020;129(4):980-991. doi:10.1152/japplphysiol.00022.2020
Wrong intervention	Myint MW, Wu J, Wong E, et al. Clinical benefits of oral nutritional supplementation for elderly hip fracture patients: a single blind randomised controlled trial. Age Ageing. 2013;42(1):39-45. doi:10.1093/ageing/afs078
Wrong outcome	Neumann M, Friedmann J, Roy MA, Jensen GL. Provision of high-protein supplement for patients recovering from hip fracture. Nutrition. 2004;20(5):415-419. doi:10.1016/j.nut.2004.01.004
Wrong outcome	Olofsson B, Stenvall M, Lundström M, Svensson O, Gustafson Y. Malnutrition in hip fracture patients: an intervention study. J Clin Nurs. 2007;16(11):2027-2038. doi:10.1111/j.1365-2702.2006.01864.x
Wrong population	Patursson P, Møller G, Thomsen BB, et al. Effects of Postdischarge High-Protein Oral Nutritional Supplements and Resistance Training in Malnourished Surgical Patients: A Pilot Randomized Controlled Trial. Nutrients. 2022;14(13):2599. Published 2022 Jun 23. doi:10.3390/nu14132599
Wrong study design	Porter KH, Johnson MA. Dietary protein supplementation and recovery from femoral fracture. Nutr Rev. 1998;56(11):337-340. doi:10.1111/j.1753-4887.1998.tb01672.x
Wrong study design	Ratchford SM, Bailey AN, Senesac HA, et al. Proteins regulating cap-dependent translation are downregulated during total knee arthroplasty. Am J Physiol Regul Integr Comp Physiol. 2012;302(6):R702-R711. doi:10.1152/ajpregu.00601.2011
Wrong population	Schmid S, Blobner M, Haas B, et al. Perioperative multi-system optimization protocol in elderly hip fracture patients: a randomized-controlled trial. Un protocole d’optimisation périopératoire multisystémique pour les patients âgés subissant une fracture de la hanche: une étude randomisée contrôlée. Can J Anaesth. 2019;66(12):1472-1482. doi:10.1007/s12630-019-01475-9
Wrong outcome	Schürch MA, Rizzoli R, Slosman D, Vadas L, Vergnaud P, Bonjour JP. Protein supplements increase serum insulin-like growth factor-I levels and attenuate proximal femur bone loss in patients with recent hip fracture. A randomized, double-blind, placebo-controlled trial. Ann Intern Med. 1998;128(10):801-809. doi:10.7326/0003-4819-128-10-199805150-00002
Wrong study design	Schurch, Marc-Andre, et al. Protein supplements increase serum insulin-like growth factor-I levels and attenuate proximal femur bone loss in patients with recent hip fracture: a randomized, double-blind, placebo-controlled trial. Annals of internal medicine 128.10 (1998): 801-809.
Wrong intervention	Sullivan DH, Nelson CL, Bopp MM, Puskarich-May CL, Walls RC. Nightly enteral nutrition support of elderly hip fracture patients: a phase I trial. J Am Coll Nutr. 1998;17(2):155-161. doi:10.1080/07315724.1998.10718741
Wrong intervention	Sullivan DH, Nelson CL, Klimberg VS, Bopp MM. Nightly enteral nutrition support of elderly hip fracture patients: a pilot study. J Am Coll Nutr. 2004;23(6):683-691. doi:10.1080/07315724.2004.10719410
Wrong outcome	Tengstrand B, Cederholm T, Söderqvist A, Tidermark J. Effects of protein-rich supplementation and nandrolone on bone tissue after a hip fracture. Clin Nutr. 2007;26(4):460-465. doi:10.1016/j.clnu.2007.03.007
Wrong study design	Thomas S, Halbert J, Mackintosh S, et al. Walking aid use after discharge following hip fracture is rarely reviewed and often inappropriate: an observational study. J Physiother. 2010;56(4):267-272. doi:10.1016/s1836-9553(10)70010-2
Wrong study design	Thomas SK, Humphreys KJ, Miller MD, et al. Individual nutrition therapy and exercise regime: a controlled trial of injured, vulnerable elderly (INTERACTIVE trial). BMC Geriatr. 2008;8:4. Published 2008 Feb 26. doi:10.1186/1471-2318-8-4
Wrong outcome	Tidermark J, Ponzer S, Carlsson P, et al. Effects of protein-rich supplementation and nandrolone in lean elderly women with femoral neck fractures. Clin Nutr. 2004;23(4):587-596. doi:10.1016/j.clnu.2003.10.006
Wrong outcome	Tkatch L, Rapin CH, Rizzoli R, et al. Benefits of oral protein supplementation in elderly patients with fracture of the proximal femur. J Am Coll Nutr. 1992;11(5):519-525. doi:10.1080/07315724.1992.10718256
Wrong study design	Tomás CC, Oliveira E, Sousa D, et al. Proceedings of the 3rd IPLeiria's International Health Congress : Leiria, Portugal. 6-7 May 2016. BMC Health Serv Res. 2016;16 Suppl 3(Suppl 3):200. Published 2016 Jul 6. doi:10.1186/s12913-016-1423-5
Wrong intervention	Villani AM, Crotty M, Cameron ID, et al. Appendicular skeletal muscle in hospitalised hip-fracture patients: development and cross-validation of anthropometric prediction equations against dual-energy X-ray absorptiometry. Age Ageing. 2014;43(6):857-862. doi:10.1093/ageing/afu106
Wrong study design	Villani AM, Miller MD, Cameron ID, Kurrle S, Whitehead C, Crotty M. Development and relative validity of a new field instrument for detection of geriatric cachexia: preliminary analysis in hip fracture patients. J Cachexia Sarcopenia Muscle. 2013;4(3):209-216. doi:10.1007/s13539-013-0108-8
Wrong study design	Volkmer B, Sadler E, Lambe K, et al. Orthopaedic physiotherapists' perceptions of mechanisms for observed variation in the implementation of physiotherapy practices in the early postoperative phase after hip fracture: a UK qualitative study. Age Ageing. 2021;50(6):1961-1970. doi:10.1093/ageing/afab131
Wrong study design	Wyers CE, Breedveld-Peters JJ, Reijven PL, et al. Efficacy and cost-effectiveness of nutritional intervention in elderly after hip fracture: design of a randomized controlled trial. BMC Public Health. 2010;10:212. Published 2010 Apr 27. doi:10.1186/1471-2458-10-212
Wrong study design	Wyers CE, Reijven PL, Evers SM, et al. Cost-effectiveness of nutritional intervention in elderly subjects after hip fracture. A randomized controlled trial. Osteoporos Int. 2013;24(1):151-162. doi:10.1007/s00198-012-2009-7
Wrong outcome	Wyers CE, Reijven PLM, Breedveld-Peters JJL, et al. Efficacy of Nutritional Intervention in Elderly After Hip Fracture: A Multicenter Randomized Controlled Trial. J Gerontol A Biol Sci Med Sci. 2018;73(10):1429-1437. doi:10.1093/gerona/gly030
Wrong intervention	Wyers, C. E., et al. PP063-SUN EFFECT OF NUTRITIONAL INTERVENTION ON NUTRITIONAL INTAKE AND STATUS IN HIP FRACTURE PATIENTS: A MULTICENTRE RANDOMISED CONTROLLED TRIAL (RCT). Clinical Nutrition Supplements 1.7 (2012): 50-51.
Wrong intervention	Wyers, C. E., et al. PP065-SUN EFFECT OF NUTRITIONAL INTERVENTION ON LENGTH OF STAY, POSTOPERATIVE COMPLICATIONS, FUNCTIONAL STATUS AND MORTALITY IN HIP FRACTURE PATIENTS: A MULTI-CENTRE RANDOMISED CONTROLLED TRIAL (RCT). Clinical Nutrition Supplements 1.7 (2012): 51.
Wrong intervention	Wyers, C., et al. PP060-SUN COST-EFFECTIVENESS OF NUTRITIONAL INTERVENTION IN HIP FRACTURE PATIENTS: A MULTI-CENTRE RANDOMISED CONTROLLED TRIAL (RCT). Clinical Nutrition Supplements 1.7 (2012): 49.
Wrong study design	Xu H, Liu L, Xie J, Wang D, Huang Z, Zhou Z. A pre-operative high-protein diet can improve the serum albumin levels of patients undergoing total knee arthroplasty. Chin Med J (Engl). 2023;136(4):491-493. Published 2023 Feb 20. doi:10.1097/CM9.0000000000002209
Wrong intervention	Yap, K. S., et al. The Feasibility and Benefits of Preoperative Whey Protein-Infused Carbohydrate Loading in Elderly With Hip Fractures Undergoing Surgery: A Pilot Study. Geriatric Orthopaedic Surgery & Rehabilitation (2021).
Wrong population	Zhang X, Huang H, Yu Y, Yang J, Liang Z, Chang C. Impact of whey protein isolate and eccentric training on quadriceps mass and strength in patients with anterior cruciate ligament rupture: A randomized controlled trial. J Rehabil Med. 2020;52(3):jrm00035. Published 2020 Mar 31. doi:10.2340/16501977-2664
